# White People

**Published:** 1874-10

**Authors:** 


### WHITE PEOPLE.

Vossius estimated that there were thirty millions of white people in Europe in 1650. At that time Great Britain and Ireland had only two millions of inhabitants. In seventy-five years this population was doubled. In 1870 the population of Europe was three hundred millions, and no doubt it will be five hundred millions in fifty years, Gibbon was afraid that all Europe would be overrun by Asiatics, and the whites driven off to the countries beyond the Atlantic; but everything seems to show that the Caucasian or white race will overrun the globe, instead of occupying a narrow run of country in the old world, as they did two hundred years ago. Great Britain and America, with their intellect, their energy, and their religion, are destined to give their language to this planet, and with it their civilization, their churches and their schools, and thus shape the destinies of the world. An illustration of the "survival of the fittest."

